# Network analysis of common genes related to esophageal, gastric, and colon cancers 

**Published:** 2017

**Authors:** Padina Vaseghi Maghvan, Mostafa Rezaei-Tavirani, Hakimeh Zali, Abdolrahim Nikzamir, Saeed Abdi, Mahsa Khodadoostan, Hamid Asadzadeh-Aghdaei

**Affiliations:** 1 *Faculty of Paramedical Sciences, Shahid Beheshti University of Medical Sciences, Tehran, Iran*; 2 *Proteomics Research Center, Faculty of Paramedical Sciences, Shahid Beheshti University of Medical Sciences, Tehran, Iran*; 3 *Proteomics Research Center, Department of Tissue engineering and Applied Cell, School of Advanced Technologies in Medicine, Shahid Beheshti University of Medical Sciences, Tehran, Iran*; 4 *Faculty of Medicine, Shahid Beheshti University of Medical Sciences, Tehran, Iran*; 5 *Gastroenterology and Liver Diseases Research Center, Research Institute for Gastroenterology and Liver Diseases, Shahid Beheshti University of Medical Sciences, Tehran, Iran*; 6 *Department of Gastroenterology and Hepatology, Isfahan University of Medical Sciences, Isfahan, Iran*; 7 *Basic and Molecular Epidemiology of Gastrointestinal Disorders Research Center, Research Institute for Gastroenterology and Liver Diseases, Shahid Beheshti University of Medical Sciences, Tehran, Iran *

**Keywords:** Colon cancer, Gastric cancer, Esophageal cancer, Gene ontology, Biomarker

## Abstract

**Aim:**

The aim of this study was to provide a biomarker panel for esophageal, gastric and colorectal cancers. It can help introducing some diagnostic biomarkers for these diseases.

**Background:**

Gastrointestinal cancers (GICs) including esophageal, gastric and colorectal cancers are the most common cancers in the world which are usually diagnosed in the final stages and due to heterogeneity of these diseases, the treatments usually are not successful. For this reason, many studies have been conducted to discover predictive biomarkers.

**Methods:**

In the present study, 507 genes related to esophageal, gastric and colon cancers were extracted.. The network was constructed by Cytoscape software (version 3.4.0). Then a main component of the network was analyzed considering centrality parameters including degree, betweenness, closeness and stress. Three clusters of the protein network accompanied with their seed nodes were determined by MCODE application in Cytoscape software. Furthermore, Gene Ontology (GO) analysis of the key genes in combination to the seed nodes was performed.

**Results:**

The network of 17 common differential expressed genes in three esophageal, gastric and colon adenocarcinomas including 1730 nodes and 9188 edges were constructed. Eight crucial genes were determined. Three Clusters of the network were analyzed by GO analysis.

**Conclusion:**

The analyses of common genes of the three cancers showed that there are some common crucial genes including TP53, EGFR, MYC, AKT1, CDKN2A, CCND1 and HSP90AA1 which are tightly related to gastrointestinal cancers and can be predictive biomarkers for these cancers.

## Introduction

 Gastrointestinal cancers (GICs) including esophageal, gastric and colorectal cancers, are the most common cancers in the world, especially in developed countries (1). Esophageal cancer is the eighth common cancer-related deaths in the world which caused death of more than 400,000 patients until 2005. In the last decade, opposite of colon, breast, lung and prostate cancers, the rate of esophageal cancer has risen rapidly (2, 3). Gastric cancer is the second common cancer-related deaths in the world. Although the mortality rate of the cancer has been decreased in recent years, the prevalence of gastric and gastroesophageal cancers have risen suddenly (4).

 Colorectal cancer (CRC) is the third most frequently diagnosed cancer in males and the second in females. One of the main risk factors of these diseases in the western countries is the life style (5).Despite a wide range of studies about these diseases to determine biomarkers, extensive heterogeneity of patients has led to the failure of target therapy. The late diagnosis in acute stage of the disease will lead to an increase in the mortality rate (6). Association of these cancers with chronic digestive problems such as intestinal polyps and chronic injuries in the esophagus and stomach tissues, as well as the significant relationship between these three cancers implies the common predictive biomarkers to investigate (7-8). 

Bioinformatics approaches specially study of protein networks of diseases have attracted attention of many medical scientists. So far, there are many bioinformatics studies on various diseases to determine diagnostic and therapeutic biomarkers. Some of these studies have been done on the neurodegenerative diseases such as MS, Alzheimer's and Huntington's (9-10). In addition, some other studies on diseases like depression and psychiatric disorders and some cancers have been conducted (11-13). This approach can lead to provide new diagnostic protocol for early detection and prognosis of these three diseases (14).

**Table 1:** Common differential expression genes in esophageal, gastric and colon adenocarcinomas

Description	Name of gene	R
RAC-alpha serine/threonine-protein kinase	AKT1	1
G1/S-specific cyclin-D1	CCND1	2
Cadherin-1	CDH1	3
Cyclin-dependent kinase inhibitor 2A	CDKN2A	4
Homeobox protein CDX-2	CDX2	5
Epidermal growth factor receptor	EGFR	6
Receptor tyrosine-protein kinase erbB-2	ERBB2	7
Gastrin	GAST	8
Interleukin-1 beta	IL1B	9
Keratin, type II cytoskeletal 7	KRT7	10
Mucin-2	MUC2	11
Mucin-5AC	MUC5AC	12
Myc proto-oncogene protein	MYC	13
Prostaglandin G/H synthase 2	PTGS2	14
Single-strand selective monofunctional uracil DNA glycosylase	SMUG1	15
Cellular tumor antigen p53	TP53	16
Thymidylate synthase	TYMS	17

Protein-protein interaction (PPI) is one of the most fundamental underlying mechanisms of life. PPI network analysis is an attractive field in proteomics and bioinformatics which provides deeper understanding of cellular and molecular processes in the case of diseases (15). Any disturbance in protein interactions can cause the onset of a disease. Many human diseases are the result of such disorders (16). PPI network analysis accompanied with GO analysis can be considered as an excellent complementary research for experimental studies about mechanisms and risk factors of diseases, drug resistant mechanism and detection of predictive and therapeutic biomarkers (17). Gastrointestinal cancers are the most common related deaths in the world and early diagnosis can help better targeted therapy and reducing mortality of patients. So bioinformatics analyses can be one of the solutions to these cancers (18).

**Figure 1:**
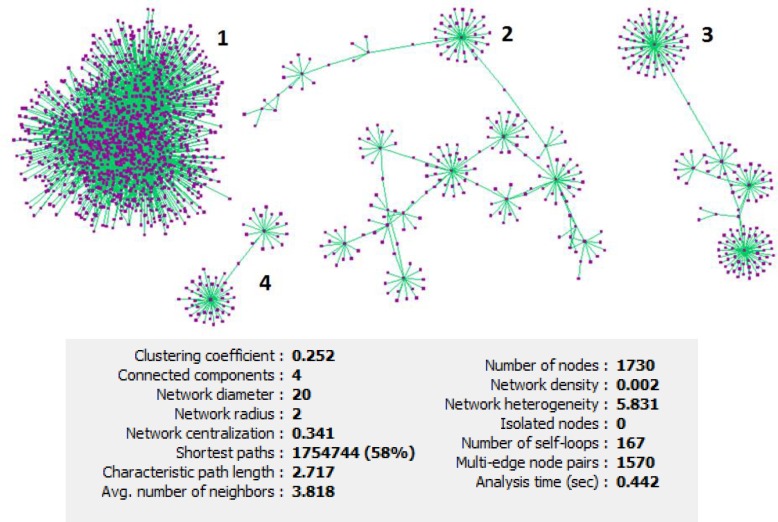
Protein-protein interaction network of esophageal, gastric and colon adenocarcinomas included 1730 nodes and 9188 edges. The network included four connected components characterized by 1297, 212, 160 and 61 nodes, and 8323, 425, 304 and 136 edges, respectively. Statistic of the network has been shown at the bottom of the figure

In this research, the common genes involved in esophageal, gastric and colorectal cancers are interacted to achieve a suitable biomarker panel. 

## Methods

Esophageal, gastric and colon cancers-related genes were extracted from String database. Of the 507 genes, 232, 143 and 132 genes were related to colon, gastric and esophageal cancer, respectively. 17 common genes were identified between these cancers (Table 1). Then protein network of these proteins was created using Cytoscape software (version 3.4.0). A main component of the network was selected and analyzed considering centrality parameters including degree, betweenness, closeness and stress. According to numerous literatures, centrality analysis is a useful method for ranking of network elements. This analysis can identify the key players in biological processes (19). Suitable cutoff for degree values was determined by average of degree plus two standard deviations. The top 5% genes based on betweenness centrality (BC), closeness Centrality (CC) and stress were selected for more analyses. The crucial genes were highlighted. Three clusters of the protein network’s main component accompanied with their seed nodes were determined by MCODE application of Cytoscape software. Furthermore, Gene Ontology (GO) analysis of the key genes in combination to the seed nodes was performed. Molecular function, cellular components and biological process were analyzed by ClueGO application of Cytoscape software. In addition, the pathways related to the proteins of each cluster checked out by KEGG database. 

## Results

**Figure 2:**
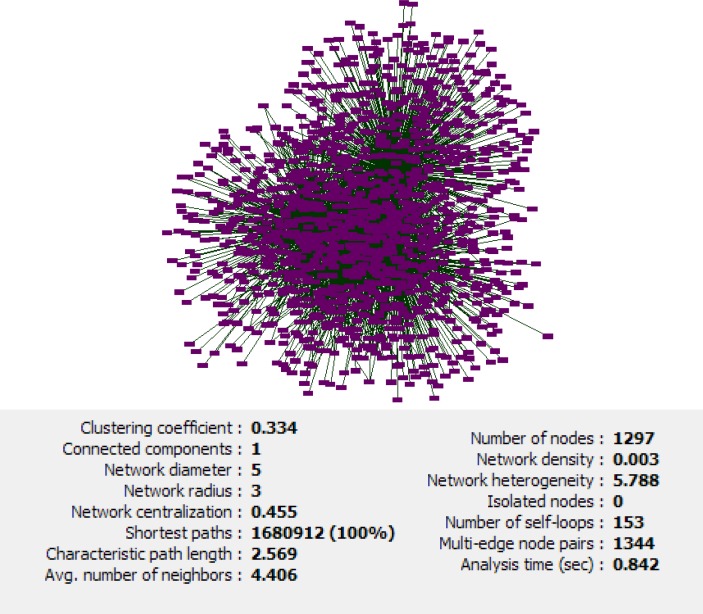
Main connected components of protein-protein interaction network in esophageal, gastric and colon adenocarcinomas including 1297 nodes and 8323 edges with the statistical information

**Figure 3:**
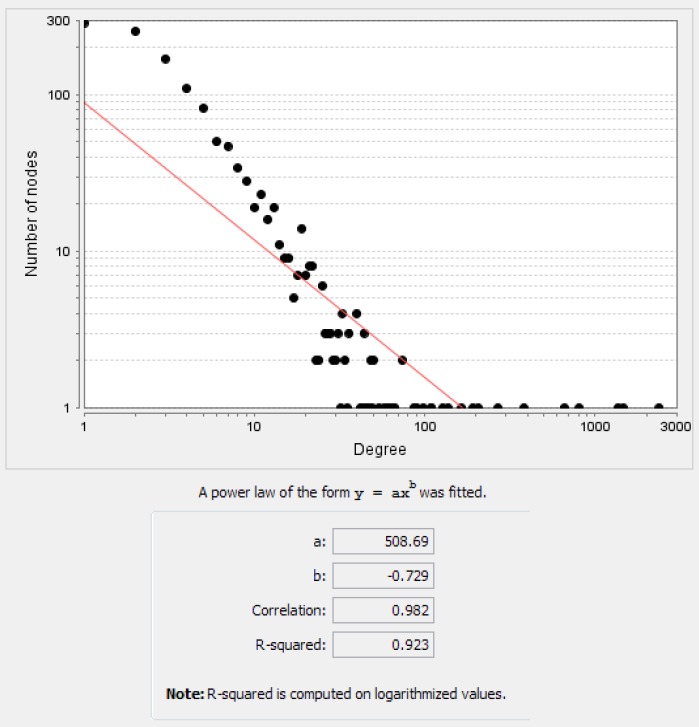
Degree distribution for the nodes of main connected components. The equation and the other statistical parameters of power law have been shown at the bottom of the figure. Closed correlation in degree values of the nodes is corresponded to the scale free network

The network of 17 common differential expressed genes in esophageal, gastric and colon adenocarcinomas including 1730 nodes and 9188 edges was constructed (Figure 1). This network contains four connected components. Network of the main connected components and the related statistical information have been displayed in Figure 2. Closed correlation in degree values of the nodes is corresponded to the scale free network (Figure 3). According to cutoff value, eight proteins with the highest degrees were selected as hub nodes of the network. All of these proteins were bottleneck nodes. All hub-bottleneck nodes were included in the selected nodes based on stress. Seven of hub-bottleneck nodes were presented in the identified nodes considering closeness (see Table 2). The presence of hub-bottlenecks in the determined clusters was investigated. In cluster 1 which has the highest score, there were three hub-bottlenecks (AKT1, TP53 and MYC). Moreover, one of hub-bottlenecks was in cluster 2 (CDKN2A) and three hub-bottlenecks were in cluster 3 (HSP90AA1, CCND1 and EGFR). None of the crucial proteins was common between the analyzed clusters. The clusters 1 and 3 were introduced as important protein complexes related to the studied cancers. The seeds of clusters 1-3 are SP1, TUBA1A and HDAC2. 

**Figure 4:**
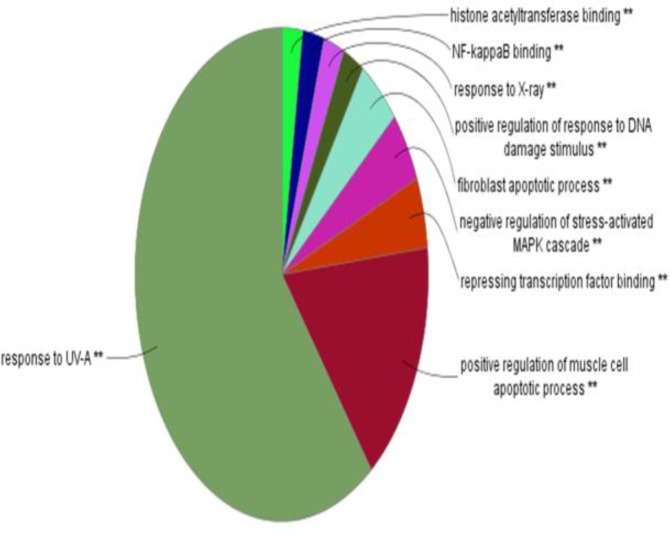
Nine clustered GO terms (the 43 presented terms in the figure 4) for the elements of main connected components

**Figure 5:**
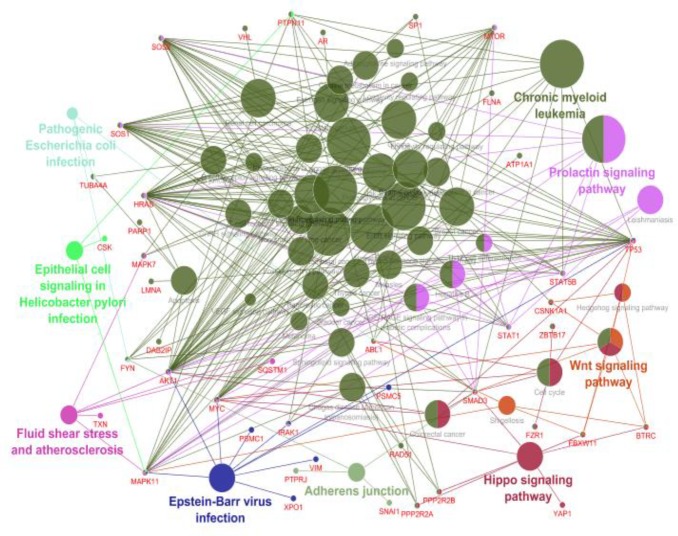
KEGG pathways related to cluster 1. Each color indicates a unique pathway. The nodes highlighted by two or more colors are corresponding to several affected pathways

**Figure 6:**
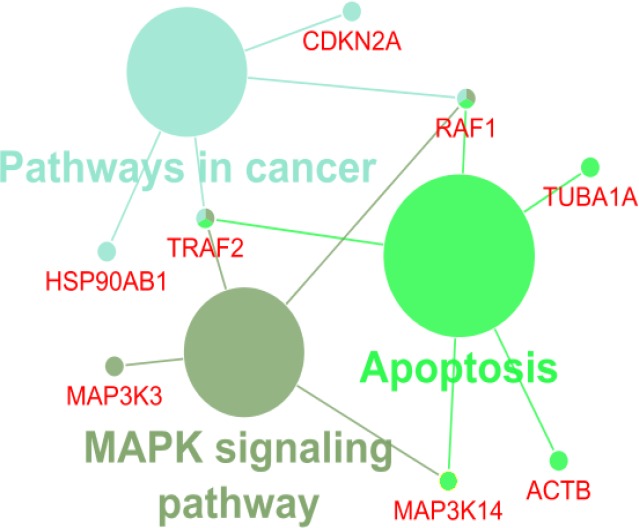
KEGG pathways related to cluster 2. The single color indicates a unique pathway. The nodes highlighted by two or more colors are corresponding to several affected pathways

**Figure 7:**
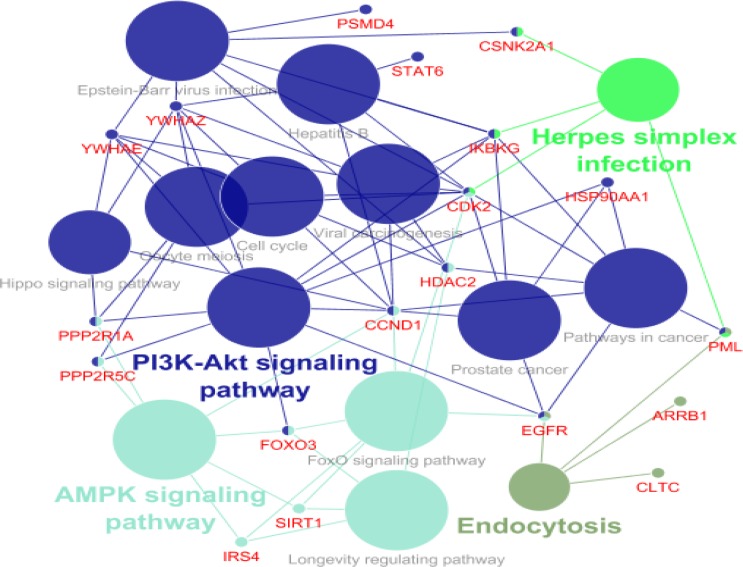
KEGG pathways related to cluster 3. The single color indicatesa unique pathway. The nodes which highlighted by two or more colors are corresponding to several affected pathways

Gene Ontology analysis of hub-bottleneck nodes and the identified seeds was performed by ClueGO application (Figures 4 and S 1). Results of cluster’s GO based on KEGG database have been shown in Figures 5-7.

**Table 2:** Common crucial genes with differential expression between esophageal, gastric and colon adenocarcinomas

Hub	Hub-bottleneck	Common proteins with top 5% nodes based on stress	Common proteins with top 5% nodes based on CC	Cluster 1	Cluster 2	Cluster 3
TP53	*	*	*	*		
EGFR	*	*	*			*
MYC	*	*	*	*		
MDM2	*	*				
AKT1	*	*	*	*		
CDKN2A	*	*	*		*	
CCND1	*	*	*			*
HSP90AA1	*	*	*			

In the first column the hub nodes have been presented. The second column shows that all hub nodes are bottleneck. The third and the fourth columns illustrate the presence of the hub-bottleneck nodes as the selected nodes based on stress and closeness centrality (CC), respectively. In the other columns, the presence of the nodes in the related clusters has been highlighted

## Discussion

In the biological network, nodes can be genes, proteins, metabolites or diseases, and edges indicate the relationship between nodes. The hubs are nodes that have the most connection with the nodes around them. Hub-bottleneck nodes are the hub nodes that have more control role on the other nodes of network (20). Most biological networks are scale-free networks. That means the notion of scale-free refers to the lack of a characteristic degree or scale. In scale free networks, the most nodes have a degree close to the average (21).

In the present study, 507 genes related to esophageal, gastric and colon cancers were extracted. The network was constructed by the common genes and the crucial genes were selected based on their relationship with other nodes. Like many cancers, there is a reasonable possible biomarker panel related to the studied cancers (14, 22). The findings indicate that the network includes three distinguished clusters. 

According to KEGG database analysis, one of the key genes in cluster 1 is TP53 which plays a role in WNT signaling pathway (23). TP53 has an important effect on the cell cycle and prolactin signaling pathway. Many studies have linked prolactin levels with the development of various forms of cancer (24, 25). In cluster 1, AKT1 is effective in WNT signaling pathway, fluid shear stress and atherosclerosis and many studies confirm these roles (26, 27). Infections caused by viruses or changes in the natural flora of the digestive tract are the important topics discussed in advanced digestive diseases (7, 28, 29). In this cluster, the CSK, MAPK11 and PTPN11 genes are involved in the epithelial cell signaling in Helicobacter pylori (H. pylori) infection. In this regard, many studies have identified the association of gastric ulcer disease with gastric cancer (30). According to GO analyses, the seed of cluster 1 (SP1) plays some roles in breast cancer, choline metabolism and GnRH signaling pathway. In recent years, attention has been paid to GnRH signaling pathway and choline metabolism for the diagnosis and treatment of cancer (31, 32). In cluster 2, the key genes CDKN2A, HSP90AB1, TRAF2 and RAF1 are effective agents in cancer pathway. In cluster 3, CCND1 and EGFR play role in MAPK signaling pathway and HSP90AA1 is important in PI3K-AKt pathway. Both of the pathways are related to the cell cycle, apoptosis, invasion and differentiation (33, 34). Moreover, some genes of cluster 3 participate in herpes simplex infection which is another indication of the role of chronic infections in the development of cancers and many studies have confirmed these evidences (35, 36).

By GO analysis, nine main processes including 43 GO terms were identified. The dominant process is “response to UV” that consists of cell transfusion, ligand binding, cell growth, and response to epidermal growth factor. The next dominant term group are focused on apoptotic processes and the regulation of the cell aging process. Regulation of fibroblastic cells and apoptosis process of them are the third and the fourth processes, respectively. Tight relationship between the components of this process and the studied cancers is confirmed by literatures (18, 37-39). From many years ago, epidermal growth factor receptor is considered as a therapeutic target for gastrointestinal cancers (40). The relationship between the cell aging and cancer has been proven by many studies (41). Aging and H. pylori inflammation as two risk factors of gastric cancer have been highlighted in the previous studies (42). Since the regulations of fibroblastic cells and their apoptotic process were introduced as the concern terms, the relationship between gastrointestinal cancers and fibroblast cells has been discussed repeatedly (43, 44).

In the present study, the analyses showed that some common crucial genes such as TP53, EGFR, MYC, AKT1, CDKN2A, CCND1 and HSP90AA1 are tightly related to gastrointestinal cancers. More analyses indicated that it is possible to introduce a biomarker panel which can be used for prognosis and early detection of gastrointestinal cancers however more validation is required.
